# Medical and Physical Intelligence

**Published:** 1801-11

**Authors:** 


### MEDICAL AND PHYSICAL INTELLIGENCE.

### [ FOREIGN AND DOMESTIC. ]

### TROMMSDORF's *Method of preparing the muriated Barytes without Kali.*

The chief point in preparing the muriated barytes confifts in feparating the fulphuric acid from the fulphat of barytes, which was formerly done by the decompofition of that acid, for which purpofe the fulphat of barytes was mixed and heated for fome time with charcoal powder, and afterwards treated with acids. In this way the coal decompofed the fulphuric acid by uniting itfelf with the oxygen, and by being difengaged under the form of carbonic acid, while the fulphur remained combined with the barytes, from which it was feparated by the addition of an acid. This method, however, was afterwards quitted, on account of the fmall quantity of falt obtained in this manner, and recourfe was had to carbonate of kali, with which the fulphat of barytes was treated in the dry as well as in the humid way. But the rifing price of that fubftance induced Mr. TROMMSDORF to try whether the old method might not be revived and rendered more profitable. With this view he undertook a feries of experiments, the refults of which are the following:

1. The decompofition of the fulphat of barytes by means of charcoal powder is very imperfect in the red heat, to which circumftance it is owing that but a fmall quantity of fulphuric acid was feparated by the former proceeding.

2. For decompofing two pounds of fulphat of barytes, a white heat, lafting about two hours, is required, though even at this degree of fire, a part of the barytes remains undecompofed.

3. Much depends on the proportion of charcoal powder that is added, and though but a fmall quantity of it is required for the decompofition of that fubftance, yet the proportion in which it was formerly taken, could never produce a perfect decompofition.

4. A too great proportion of the charcoal powder is likewife difadvantageous, becaufe the charcoal being a bad conductor of heat, prevents the whole mafs from receiving that degree of temperature which is neceffary for the decompofition.

5. A very pure muriated barytes is obtained by diffolving the above mixture in pure muriatic acid, which is not the cafe if it be impregnated with fulphurous particles.

6. Pure barytes poffeffes a great affinity to the fulphur, as the moft intenfe heat is not capable of feparating them.

The proceeding itfelf is as follows: Take two pounds of pure white fulphat of barytes, heat them in a crucible till they fall into a white a white powder, and when they become cool, make the powder ftill finer by trituration. In cafe the fulphat of barytes fhould contain any iron, digeft it with muriatic acid, by which means the trouble of purifying the muriated barytes will be afterwards faved. Mix the above quantity of finely pulverifed fulphat of barytes with three ounces of very finely pulverifed charcoal which has been previoufly heated, and put the whole into a ftrong crucible that is provided with a cover, the junctures of which, with the crucible, ought to be carefully fecured with lute, which having become dry, the veffel is placed into a furnace for fufion. Let the fire be gentle at firft, but increafe it by degrees, till the veffel becomes white hot, in which ftate it muft be kept for two hours. On opening the veffel, when it becomes cool, the mafs will be found melted, or rather baked for the moft part. It is now pulverized in a ftone mortar, and infufed with diftilled water till it gets the confiftency of an uniform mafs. Add to this very diluted pure muriatic acid, which continue as long as any effervefcence is to be perceived; all the time a great quantity of fulphurated hydrogen gas will be evolved, and the mixture rife up confiderably. The liquor is then heated till it begins boiling, and afterwards cooled and filtrated. On evaporating it, a white and pure muriated barytes will be obtained.

Befides, the kali, much time is alfo faved by this method of proceeding, which always fucceeds in a good furnace. Mr. Trommsdorf remarks by the way, that the method of preparing the muriated barytes by means of heating a mixture of fulphat of barytes, commou falt, and charcoal powder, propofed by Cit. *Lauaet*, does by no means yield that falt; the production thus obtained is nothing but a combination of the fulphurated hydrogen gas, fulphur, and free barytes.

---

### *Easier Mode of preparing the Martial Flowers, or the ammoniated Iron.*

It is univerfally known, that the flores ammoniaci martiales are compofed of fal ammoniæ and the muriate of iron, in which the muriatic acid is partly combined with the iron, partly with ammonia, and that this triple falt is produced by the decompofition of part of the fal ammoniæ, and that the iron is but flightly attached to it. Several modes of preparation were built on that difcovery, none of which however could be faid to be preferred as the eafieft and beft, till Mr. Schiller, an apothecary of merit, came to prepare the ammoniated iron without fublimation, which he performed by perfectly evaporating in a glafs veffel one ounce of fal ammoniæ diffolved in water, to which he had previoufly added a folution of iron in fix drachms of diluted muriatic acid. This eafy and advantageous mode of preparation was not fo much attended to as it deferved, and the neweft and beft pharmaceutical works ftill recommended the former mode of preparing that remedy by fublimation, though after different directions. But by the above method, not only much time and fire is faved, but the preparation becomes becomes always more uniform than is the case with the sublimation. The flores salis ammoniaci martiales ought therefore to be prepared by mixture and evaporation, according to the prescription of Mr. Schmidt, who takes to one ounce of sal ammoniæ dissolved in two ounces of water, one drachm of liquefied muriate of iron, and evaporates this mixture to dryness, which exhibits a substance perfectly the same as if it had been prepared by sublimation.

---

### *Examination of the colouring part of the blood, by Fourcroy and Vauquelin.*

The colour of the blood seems, in a great measure, to be owing to the iron which was first discovered in this fluid by Menghini; but in what state it existed there, had hitherto been left uncertain. Deyeux and Parmentier thought the iron to be combined in the blood with natron, in a state analogous to the tinctura alkalina. Sage and Gren imagined that it might be united with phosphoric acid; a conjecture, the truth of which is at present confirmed by the late experiments of those two great chemists, Fourcroy and Vauquelin; the results of which are the following.

The red part of the blood, precipitated from the water with which the crassamentum had been washed, being burned in a crucible, a residuum of a dark red colour remained, which contained iron, and amounted to about 0,0045 of the blood employed in the examination. For separating the iron, or its natural combination, from the adherent salts and other heterogeneous particles, the above residuum is digested in diluted nitric acid, in which a part of it dissolves, while another remains undissolved, and obtains a high red colour. Ammonia occasions in this solution a precipitation of phosphat of kali. Muriatic acid may be likewise employed for dissolving the phosphat of iron, that remains after having heated the above residuum. The phosphat of iron appears in a double state, according to the degree of its oxydation, in the first of which it is of a greyish colour, of a glossy surface, not soluble in water, but soluble in acids; whereas in the other state it is red or brown, and less soluble in acids than in water; in the first state the phosphoric acid is exactly saturated with iron, while in the other case it is supersaturated with the oxyd of iron. Caustic alkalis do not entirely decompose the white phosphat of iron, but only deprive it of a part of the acid, leaving the phosphat of iron in a state of supersaturation; in which it exists in the blood, dissolved by the serum, and the blood of all animals is coloured by the phosphat of iron supersaturated with oxyd. In this state it dissolves easily in the white of an egg and in serum, when it is shaken with these fluids, by which a red solution will be obtained, similar to blood. The presence of natron in the blood contributes greatly to produce that state of the phosphat of iron, and at the same time heightens the colour of it.

It is a characteristic property of the red serum, that its colour is changed changed by the contact with air, as it becomes light red in oxygen gas, and dark red or brown in carbonic, but particularly in hydro-carbonic gas. It likewife acts on the atmofphere by imbibing its oxygen, and forming carbonic acid.

All thefe phenomena feem undoubtedly to depend on the phofphate of iron, becaufe they manifeft themfelves particularly when it is united with the ferum.

It is obferved by *Vauquelin*, that the colouring part of the blood diffolves copper with great eafe, becaufe the refiduum of the ferum fanguinis, which had been boiled in a clean veffel of copper, in order to coagulate the albuminous particles, fhowed evident traces of its containing copper. On precipitating its folution in muriatic acid by ammonia, the liquor received a beautiful blue colour, which difappeared again on adding more muriatic acid. An iron plate put into the folution was immediately covered with copper particles. Mr. Vauquelin, however, is of opinion that this folution of copper proceeds particularly from the albuminous matter, as the liquor obtained by wafhing the craffamentum fanguinis with water, did not diffolve copper, when previoufly freed from the albuminous particles. We ought therefore to take care not to boil blood which is intended for food, in copper veffels. The liquor obtained by wafhing the craffamentum fanguinis, confifts accordingly of much water, albuminous and gelatinous fubftance, and phofphat of iron, which being diffolved in the albuminous matter is precipitated with it, as foon as it coagulates. The albuminous matter however, notwithftanding its combination with the phofphat of iron, acts on the copper, and probably alfo on other metals. The above liquor contains likewife natron and fome other falts. The co-exiftence of the phofphat of iron and of natron is not at all furprifing, when we confider that fixed alkalis do not entirely decompofe the phofphat of iron, but change it into a phofphat with fuperabundance of oxyd, in which ftate it exifts in the blood. It is likewife known that iron is oxydated in a folution of phofphat of natron, and by decompofing this falt for the moft part becomes a phofphat of iron. It is probable that this fubftance is formed in that manner in the blood, whereby at the fame time the natron is difengaged.

---

*On the Marrow of Bones, by Isenflamm.*

The fat, oily, and tranfparent fubftance contained in the bones of animals, particularly of the mammalia, is called Marrow, which appears in fome animals more, in others lefs fluid during life, but is always found folid after death. It is moft frequently of a yellowifh colour; in the long bones it has greater confiftency, but is more fluid and of a reddifh colour in the polyedrous and flat bones, and at both ends of the long bones, to which it alfo imparts a darker colour. The folid fubftance of the bones is likewife penetrated by the fineft marrow. The cavities and cells of the os frontis, os cribriforme, os fphenoideum, and maxilla fuperior, are without without it, and appointed to different purpofes. Some fpecies of birds are provided with bones, which, inftead of marrow, are filled with air, analogous to the proceffus maftoideus of the temporal bones, whofe cells communicate with the tuba Euftachii by means of the cavum tympani. The marrow is enclofed by the perioftium internum, or the marrow membrane, and it appears under the microfcope like an accumulation of globules, the diameter of each of which is between 0,08 and 0,06 of an inch. It is not tinged by madder taken internally, as no fuch effect was produced in two dogs that were fed for fome time with that root, although their teeth were tinged of a deep red colour. The conftituent particles of the marrow are the fame as other fats, namely, oil, water, and acid; it contains no alkali, on which account it is lefs liable to putrefaction, though it becomes rancid when expofed to the contact of air, and changes its colour. Its fpecific weight is greater than that of water, though Plenk and Soemmering think it is lighter. It is fecerned from the blood by arteries, which penetrate into the bones, moft probably from their ends, and not by tranfudation. In confumptive difeafes it is reforbed by the lymphatic veffels, but foon reftored when thofe difeafes are over. It is entirely deprived of fenfibility, like all liquors fecerned from the blood. In the embryo and the infant child, it appears in form of a deep red jelly, furnifhed with a great quantity of blood-veffels; but when age is more advanced, it obtains a greater confiftency, the blood-veffels decreafe, and the colour becomes yellow. In old people it is generally dark yellow, and folid.

It feems to be more folid and yellow in the male than in the female fex, the latter poffeffing likewife a lefs proportion of it. Horfes have proportionably lefs of it than men, their cylindrical bones being much narrower and more folid than in men. The globules of the marrow of horfes appear fmaller under the microfcope than thofe of the human body, and are alfo whiter, and of a more fluid confiftency. In oxen it appeared white and folid, fomewhat like tallow; but in calves it is of a reddifh colour, and fluid like jelly. In fheep it is more fluid than in men, and appears like oil coagulated by cold. In dogs its colour was more red, and of a greater confiftency than in men. In a puppy of fome weeks old it appeared as a reddifh mucilage. In a goofe it was of a light yellow, and fluid, as in men. In a cock it appears as a deep red jelly; in the hen, however, it is yellowifh and folid. In the hog it is reddifh, and more confiftent than in men. In the whale it is very fluid.

The marrow feems not to be expofed to idiopathic difeafes, but is only changed when the bones are affected at the fame time. Mr. Troja obferved, that it became white, folid, and ready to offify in the upper part of a broken bone; and Mr. Boon found it fpongy and fibrous in the callus of broken bones; but in the mollities offium it appeared, according to the obfervations of Navier, like fluid fat.

The ufe of the marrow for animal economy has been much difcuffed by phyfiologifts, but all opinions hitherto brought forward on this fubject feem to be expofed to doubts and exceptions. It has been thought by fome naturalifts to contribute to the formation and growth of the bones, which however is contradicted by the obfervation that the cartilaginous fubftance of which the bones confift in the embryo and infant are quite deprived of marrow, and likewife the bones of birds, &c. Hippocrates and Galen were of opinion that it might ferve for the nutrition of the bones, which however is alfo oppofed by the above obfervation. Others think it of great fervice in producing the callus and reftoring the bone; but it may be fuggefted, that fractures of the bones are healed in a much fhorter time the lefs the patient is advanced in years, and particularly at a period when the marrow is not yet perfectly formed. Mr. Troja and Blumenbach remarked befides, that notwithftanding they purpofely deftroyed the marrow, the bone was reproduced. Another opinion, adopted by the moft refpectable phyfiologifts, as Bertin, Haller, Blumenbach, &c. is, that it renders the bones flexible, and communicates to them ftrength and elafticity. In order to prove this opinion, Bertin fuggefts, that the bones, deprived of their marrow by fire, became brittle and eafily to be fractured; whereas, when put into oil for fome time, they would recover their former flexibility. But this experiment frequently proved of a contrary effect; and befides, we remark, that the bones of children, in which the marrow is not yet perfectly formed, poffefs the higheft degree of flexibility. Several phyfiologifts have imagined that the marrow may ferve for anointing the joints, an opinion particularly maintained by Havers; but the exiftence of the pores, through which it was thought to get to the joints, though defcribed by Havers, has not been afcertained, and the above function feems to be fulfilled by the fynovia. Mr. Soemmering ingenioufly conjectures, that the marrow is only intended to fill the vacuum in the bones, every other fubftance being too heavy for that purpofe. It is however moft probable, that it is intended by Nature for different purpofes, according to the different ages of the animal. If we are allowed to conclude from the ufe of the reft of fat, we imagine that its principal ufe in old people confifts in confining heat; for we know that fat has the property of preferving heat, on which account fat people feel the cold lefs than lean people, and the fecretion of fat proceeds much better in winter than in fummer. For the fame reafon, the marrow is moft likely in fuch quantity and perfection in old people, in order to preferve the heat internally, becaufe lefs fat is depofited in the external parts at an old age. Lean people frequently complain of a fenfation of cold in the bones, and the fame may be obferved in perfons affected with confumptions, who, though almoft confumed by a burning heat in the external parts, have a fenfation of cold internally in the limbs. In children, however, who are generally fat, it confifts of a kind of gelatina, which perhaps contributes to nutrition, and the bones may confequently be confidered, ed, at this period, as repofitories of materia nutritiva. Whether the marrow alfo binds electric matter, with which the bones are thought to be provided, muft be afcertained by farther obfervations; and it is probable that the examination of bodies of people who have been killed by lightning, may throw fome light upon this fubject.

---

We acquainted our readers in a former number of this Journal, with the properties of a new earth, difcovered by Profeffor Trommsdorf of Erfurt, in the Saxon beryll, and ftyled by him *Agust earth.* In his former analyfes, he found that this foffil contained at the fame time filiceous and aluminous earth, which particles, however, he was inclined to think adventitious, and perhaps originating from the matrix in which that foffil occurs. In order to afcertain this he undertook a new analyfis, for which purpofe he endeavoured to obtain the cryftals of the foffil in as pure a ftate as poffible, and free from any adherent particles; and though this proved very difficult, on account of their minutenefs, he fucceeded in feparating 100 grains of very pure cryftals, which were of a fky blue colour. According to the experiments he made with them, no aluminous earth was to be difcovered; but 100 parts of the Saxon beryll or aguft it confifted of

| | |
|---|---|
| 1,0 | water |
| 1,0 | filiceous earth |
| 1,5 | oxyd of iron |
| 95,0 | aguft earth |
| 1,5 | lofs |
| 100 | |

The oxyd of iron and the filiceous earth appear only adventitious particles.

The feparation of the cryftals is attended with fo much difficulty, that Mr. Trommsdorf thought of an expedient for feparating the pure aguft earth from the whole foffil, in which the aguft lay difperfed. The foffil was accordingly very finely pulverized and boiled with muriatic acid of middle ftrength, till a white powder only remained, confifting of filiceous earth. The liquor, when filtrated, contained oxyd of iron and of manganefe, aguft earth, argillaceous earth, and a little magnefia. A fufficient quantity of pruffiat of kali having been added to it, it was heated to the boiling point, whereby the metallic particles and a part of argillaceous earth were feparated in combination with the pruffic acid. The remaining liquor was again boiled, and precipitated with cauftic ammonia. The precipitation being feparated by the filtre and carefully wafhed, was boiled with cauftic kali, and diffolved in pure muriatic acid, after being previoufly edulcorated. It is then precipitated by carbonat of kali, and after being frequently edulcorated and dryed, it exhibits the pure aguft-earth.

---

The

The ſame gentleman has made the following remark on the ſeparation of the argillaceous earth from the oxyd of iron by means of cauſtic lye; a moſt excellent ſubſtance for the examination of foſſils, with the uſe of which, in the analyſis of mineral bodies, we have been acquainted by the celebrated KLAPROTH of Berlin. In employing however the cauſtic lye for the ſeparation of argillaceous earth from the oxyd of iron, we ought, 1. Never to boil the argillaceous earth, which contains the iron, in glaſs or porcelain veſſels, but in ſilver ones, for fear of ſeparating the ſiliceous earth from thoſe veſſels.

2. A great quantity of cauſtic lye muſt be employed, and the whole mixture evaporated to perfect dryneſs, after which it muſt be frequently waſhed with diſtilled water. The oxyd of iron does not frequently prevent the cauſtic kali from affecting the argillaceous earth, on which account the oxyd ought again to be examined.

---

Mr. KRUGER of Roſtock, recommends the following method of preparing the concentrated vinegar. Take three ounces of vitriolum martis, and after having heated it, pulverize it with half an ounce of ſaccharum ſaturni. Put this mixture into a retort, but take care that nothing adheres to the ſides of it; expoſe it to a ſtrong fire. The quantity of acetic acid that will be obtained in this way, amounts to about two drachms. Though Prof. LOUIT's method is more productive, as he obtained about 64 parts of acetic acid from 96 parts of acetite of natron, whereas the above method yields only 48 parts of acetic acid from 96 parts of ſaccharum ſaturni, yet the ſubſtance which he uſed is more expenſive than the ſugar of lead and vitriolum martis, and, conſequently, leſs profitable.

Mr. KRUGER's method is alſo preferable to that which has been lately propoſed by Mr. PIEPENBRING, who obtained the acetic acid from the ſaccharum ſaturni by means of concentrated ſulphuric acid, but only 36 parts from 96 parts of the ſugar of lead. This acid of lead frequently ſmells of ſulphuric acid, and is impregnated with oxyd of lead. The acetic acid, however, ſeparated after the above method, is perfectly pure, neither impregnated with ſulphurous acid nor containing any particles of lead, iron, or copper.

---

The ſame gentleman propoſes a new method of preparing the nervous tincture of *Beſtucheff*, immediately from the ſulphat of iron, which is the following: Diſſolve 100 grains of green purified vitriol in 1000 grains of diſtilled water, filter it, and let it evaporate till about 240 grains of the liquor remain; 120 grains of this ſolution are mixed with half an ounce of ether vitrioli, and expoſed to moderate heat, in which the ether remains white, while the ſolution of iron is of a light yellow colour, which, however, diſ-

appears appears in the cold. The clear ether being poured off, is of a ſtrong aſtringent taſte. The remaining 120 grains of the ſolution of iron are mixed with one ounce of alkohol, and expoſed to a gentle digeſtion; whereby the alkohol obtained a light yellow colour, which diſappeared after it had been ſtanding in the cold for two days. A few days after, the alkohol, being decanted from the ſulphuric ſolution of iron, is mixed with the ſulphuric ether that had been firſt impregnated with iron. Upon accurate examination, the alkohol was found to contain about 30 grains of iron, and the ſulphuric ether about 24, and the whole mixture accordingly about 54 grains.

---

Profeſſor GOETTLING of Jena informs the public, that on analyſing Dr. Hahnemann's preſervative powder againſt the ſcarlet fever, he could not diſcover any traces of its containing a vegetable extract. The ſolution of it in ſpirit of wine became not at all tinged, though it was ſuffered to ſtand ſome time, which would not be the caſe, if it conſiſted of a vegetable extract. On evaporating the ſolution, the reſiduum was a cryſtalized ſalt, which by different proceedings manifeſted itſelf to be nothing but tartar.

---

Citizen BERTHOLLET has communicated his experiments on the muriatic acid, of which the reſults are the following: 1. Potaſh and metals are capable of transforming the nitric acid into muriatic acid. 2. The radical of the muriatic acid is to be ſearched in the water and the nitric acid, that is to ſay, in the oxygen, azot, and hydrogen. 3. It is very probable, that in the muriatic acid, the azote, as the baſis, is united with a portion of oxygen and hydrogen. *Berthollet* terminates his obſervations with remarking, that ſubſtances are more difficultly to be decompoſed the leſs the proportions of the component particles of which they are conſtituted.

---

The celebrated Italian chemiſt, L. BRUGNATELLI, Profeſſor of the Univerſity of Pavia, has lately diſcovered the tranſition of tartarous acid into the ſaccharic acid, which diſcovery will enable us to explain the origin of the diabetes mellitus. He has likewiſe obſerved, that gold cryſtallizes itſelf by means of electric ſparks, which cryſtals he calls *elettrati*.

---

FOURCROY and VAUQUELIN have analyſed the ear-wax, and found that it conſiſts of the following ſubſtances. 1. Of an oily matter, more reſembling the fat which is contained in the gall than any other animal fat. 2. Of an albuminous animal ſubſtance. 3. Of a colouring principle, which, by its bitter taſte and its cloſe combination with fat, comes near to the colouring principle of the bile.

---

The

The ſame chemiſts have endeavoured to employ the oxalic acid for determining the proportion of the phoſphoric acid and of lime in bones; by means of which they found, that 100 parts of the materia vitreæ of the teeth conſiſted of 43,23 parts of lime, 29,67 of phoſphoric acid, and 27,10 of gelatina and water.

---

William Blair, A. M. F. M. S. Member of the Royal College of Surgeons in London, Surgeon of the Lock Hoſpital and Aſylum, of the Finſbury Diſpenſary, and of the Bloomſbury Diſpenſary, &c. propoſes to deliver a Courſe of Clinical Lectures on the Diſeaſes and Operations of Surgery. Further particulars may be learned by applying at Mr. Blair's houſe, No. 69, Great Ruſſel Street, Bloomſbury Square, between the hours of nine and ten in the morning.

---

Mr. Ring will publiſh, in a few days, A Treatiſe on the Cow-Pox; containing the Hiſtory of Vaccine Inoculation, and an Account of the various Publications which have appeared on that Subject, in Great Britain and other Parts of the World.

---

